# A Forest‐Based Triboelectric Energy Harvester

**DOI:** 10.1002/gch2.202200058

**Published:** 2022-08-07

**Authors:** Jesper Edberg, Mohammad Yusuf Mulla, Omid Hosseinaei, Naveed ul Hassan Alvi, Valerio Beni

**Affiliations:** ^1^ Bio‐ and Organic Electronics RISE Research Institutes of Sweden Digital Systems Bredgatan 35 Norrköping SE‐602 21 Sweden; ^2^ Digital Cellulose Center Bredgatan 35 Norrköping SE‐602 21 Sweden; ^3^ Bioeconomy and Health RISE Research Institutes of Sweden Stockholm SE‐114 86 Sweden

**Keywords:** cellulose, energy harvesting, green electronics, lignin, triboelectric nanogenerators

## Abstract

Triboelectric nanogenerators (TENGs) are a new class of energy harvesting devices that have the potential to become a dominating technology for producing renewable energy. The versatility of their designs allows TENGs to harvest mechanical energy from sources like wind and water. Currently used renewable energy technologies have a restricted number of materials from which they can be constructed, such as metals, plastics, semiconductors, and rare‐earth metals. These materials are all non‐renewable in themselves as they require mining/drilling and are difficult to recycle at end of life. TENGs on the other hand can be built from a large repertoire of materials, including materials from bio‐based sources. Here, a TENG constructed fully from wood‐derived materials like lignin, cellulose, paper, and cardboard, thus making it 100% green, recyclable, and even biodegradable, is demonstrated. The device can produce a maximum voltage, current, and power of 232 V, 17 mA m^–2^, and 1.6 W m^–2^, respectively, which is enough to power electronic systems and charge 6.5 µF capacitors. Finally, the device is used in a smart package application as a self‐powered impact sensor. The work shows the feasibility of producing renewable energy technologies that are sustainable both with respect to their energy sources and their material composition.

## Graphical abstract

A triboelectric energy harvester based solely on renewable forest‐derived materials (forest‐based triboelectric nanogenerator, F‐TENG) is demonstrated for the first time. The F‐TENG shows performance comparable or superior to those previously reported for TENGs only partially based on biopolymers. The suitability of the F‐TENG technology as a self‐powered impact sensor is reported.

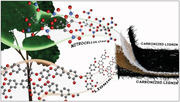

## 1 Introduction

Global climate change has reached an irreversible tipping point due to human influence. The largest contributor to this is CO_2_ emissions from burning of fossil fuels used to power our society. For over a century, nonrenewable materials like coal, oil, and natural gas have been used as our main sources of energy. The last few decades have seen upsurge in greener technologies using renewable energy sources such as wind, sun, or geothermal energy, while at the same time these are becoming cheaper to produce and easier to integrate with existing infrastructure.^[^ ^1^, ^2^ ^]^ Besides large‐scale energy generation for the power grid and automotive industry, there is also a drive to develop new energy harvesting technologies for distributed electronic devices to make them energy autonomous.^[^ ^3^, ^4^ ^]^ Triboelectric nanogenerators (TENGs) are a relatively new class of energy harvesting devices, capable of converting mechanical energy into electrical energy.^[^ ^5^ ^]^ First developed in 2012, the TENG device has proven to be a promising alternative to existing energy harvesting technologies, both on large and small scales. TENGs have been proposed to replace, or complement, existing power plants using wind and water,^[6,7]^ as well as powering wearable devices by deriving power from the body movements.^[^ ^8^ ^]^

One problem with all current energy harvesting technologies is that they are composed of nonsustainable materials like metals, plastics, and rare‐earth elements. Also, the production of technologies like solar panels requires hazardous processes, and the materials used are difficult to recycle at end of life.^[^ ^9^, ^10^, ^11^ ^]^ The smaller Internet of things (IoT) devices are becoming a part of every aspect of our society and have already started to contribute to a large amount of electronic waste, a problem that will only grow over time.^[^ ^12^, ^13^ ^]^ Among the existing energy harvesting technologies, TENGs have a unique possibility of being manufactured from a wide range of material combinations, including environmentally friendly materials derived from bioresources like wood. This is because almost all materials will have a triboelectric effect when contacted with a material with different electronegativity, and the amount of charge that will be transferred is determined by its position in the so‐called triboelectric series.^[^ ^14^ ^]^ By using forest‐based materials in electronics, these devices can become “greener” by using less energy during production and being nontoxic and partially or fully recyclable when disposed.^[^ ^15^, ^16^, ^17^ ^]^ Since wood is part of the natural CO_2_ cycle, the only emissions will come from the energy input during production. And by using nontoxic and plastic‐free materials, electronic waste will have less impact on nature, in the cases when the waste is not properly handled and ends up in nature. There have been several reports where biobased materials have been used as one or both of the triboelectric layers.^[^ ^18^, ^19^, ^20^, ^21^, ^22^, ^23^, ^24^, ^25^ ^]^ However, most often, other device components like the electrically conductive electrodes as well as encapsulation materials were made from metals, plastics, or other non‐biobased sources. For example, Roy et al. used chemically modified cellulose nanofibrils (CNFs) in combination with polyvinylidene fluoride (PVDF) as the triboelectric pair, and Al‐coated polyethylene terephthalate (PET) as the electrodes,^[^ ^26^ ^]^ and Oh et al. used CNF combined with silver nanowires and barium nanoparticles together with fluorinated ethylene propylene (FEP) as the triboelectric pair, with aluminum as electrodes.^[^ ^27^ ^]^

Here, we report a TENG made entirely from wood‐derived materials, like cellulose and lignin. The charge‐generating triboelectric layers were produced from lignin and chemically modified cellulose, the charge‐collecting electrodes were made from graphitized lignin that was made conductive by high‐temperature graphitization, and the supporting and encapsulating structures were made from cardboard and paper. To the best of our knowledge, this is the first report where all TENG device components are constructed from forest biomass, making it sustainable, recyclable, and even biodegradable. Hence, we call this device the forest‐based triboelectric nanogenerator (F‐TENG). While there are several publications on cellulose or wood used in TENGs,^[^ ^28^, ^29^, ^30^, ^31^, ^32^, ^33^, ^34^, ^35^, ^36^, ^57^ ^]^ there are far fewer report of the use of lignin.^[^ ^37^, ^38^ ^]^ Lignin is the second most abundant biopolymer on the planet after cellulose which is derived as a byproduct from the pulp and paper industry. Therefore, lignin is a cheap and largely untapped natural material source. We demonstrate that nanostructuring the electrode materials in the form of fiber networks using electrospinning resulted in up to 73 times greater charge generation compared to flat/compact electrodes. The F‐TENG materials could generate enough power to drive small electronic systems and charge a supercapacitor. Finally, a fully assembled F‐TENG was used as a self‐powered impact sensor in a smart package application, transmitting data from the package to an in‐house developed app. Building fully biobased and recyclable electronics is important in order to mitigate electronic waste, especially when used in short‐lifetime applications like smart packages. By using this concept, there is no need for separating the electronics from the cardboard at end of life, since the material sources are the same.

## 2 Results and Discussion

**Figure 1:**
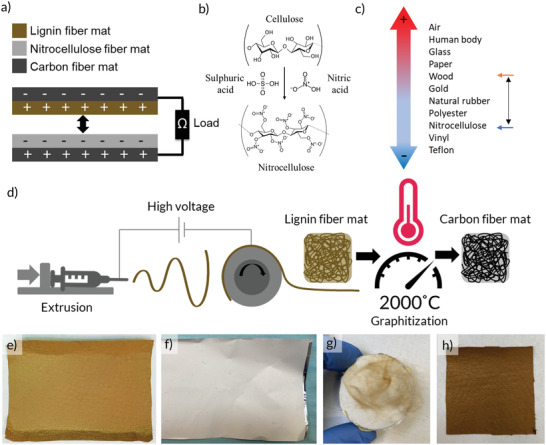
a) Schematic of the device structure and charge accumulation during operation. b) Molecular structures of cellulose and NC. c) Schematic illustration of a triboelectric series. d) Schematic of the electrospinning and graphitization process. e) Photograph of lignin fiber mat stabilized at 100 °C. f) Photograph of NC fiber mat. g) Photograph of lignin (Lig100) fibers attaching to cotton fabric after contact. h) Photograph of lignin fiber mat stabilized at 250 °C.

An all‐biomass‐derived triboelectric energy harvesting device was constructed from lignin, chemically modified cellulose (nitrocellulose), and carbon fibers formed by graphitized lignin. The amount of charge transferred in a TENG depends not only on the material properties of the triboelectric layers, but also on the contacting area. For this reason, the contact area is often maximized by different types of micro‐ or nanosurface structuring techniques.^[^ ^39^, ^40^ ^]^ In this work, each layer was produced in the form of fiber mats by electrospinning in order to achieve fibrils with diameters ranging from <100 nm up to ≈1 µm. Electrospinning is one of the simplest, versatile, efficient, scalable, and cost‐effective methods for producing sub‐micrometer fibers and high surface area nonwoven mats. This method has been successfully applied to a variety of polymers including biobased polymers like lignin and cellulose. Currently, electrospinning is finding applications in different areas such as healthcare and air filtration but also triboelectric nanogenerators.^[^ ^41^, ^42^ ^]^ The device stack is depicted in **Figure** **1**a, where lignin and nitrocellulose (NC) fiber mats constitute the positive and negative triboelectric layers, respectively, while graphitized lignin fiber mats placed on the outer sides of the device act as electrodes. NC is typically formed by treating cellulose fibers with a mixture of sulfuric and nitric acids, resulting in the substitution of hydroxyl groups with nitrate groups (Figure 1b).^[^ ^43^ ^]^ The nitrate groups make cellulose a better electron acceptor, reflected in a shift toward more negative values in the so‐called triboelectric series (Figure 1c).^[^ ^14^ ^]^ When two materials come into contact, charges are transferred between the surfaces in a process called contact electrification; the relative position of the two materials in the triboelectric series predicts the charge distribution after contact, with the materials further down in the series becoming negatively charged and with those higher up in the series becoming positively charged. Furthermore, the greater the separation between two materials in the series, the larger the charge transfer will be. Since most materials of biological origin are placed close together in the positive end of the series, it is a challenging to manufacture TENGs only from such sources due to the small charge transfer. The chemical modification of cellulose provides the necessary gap in the series to produce a charge transfer of significant magnitude to be of practical use.

Electrospinning of lignin and NC was performed by applying a high voltage between the tip of the needle and the collector plate while feeding the material in solution form (see “Experimental Section” for more details). Lignin fiber mats were further carbonized in an oven under inert atmosphere to produce carbon fibers with high conductivity (Figure 1d). Photographs of the produced lignin and NC fiber mats are shown in Figure 1e and Figure 1f, respectively. Besides the large surface area that the electrospun fiber networks provide, the use of mat‐like materials also provides elasticity/flexibility to the device, which are vital in the development of an oscillation‐based energy harvester involving repeated cycles of contact, compression, and separation. TENGs based on similar fiber networks and/or foams have been reported previously to be able to achieve high charge densities.^[^ ^44^ ^]^ However, when put in physical contact, both the lignin and NC mats proved to be weakly entangled, with fibers easily loosening and transferring to the opposite surface. As an example of this, Figure 1g shows a piece of cotton fabric brought in contact with a lignin fiber mat; significant transfer of material from the mat to the cotton was clearly observed. To enhance the interfiber connections to make the fiber mats more durable, different cross‐linking methods were employed for both the lignin and NC samples. The lignin mats were heat‐treated and compressed at different temperatures to melt and stick fibers together for producing a more interconnected fiber network. These processes, thermostabilization and compression, have been reported earlier to improve mechanical integrity.^[^ ^45^, ^46^, ^47^ ^]^ Figure 1h shows a photograph of a piece of lignin fiber mat after thermostabilization at 250 °C. Besides a slight darkening of the color, the fiber mats became significantly robust with little or no fibers detaching (see Figure SI1 in the Supporting Information). For the NC fiber mats, a novel vapor treatment was developed where the material was exposed to acetone vapor in a closed chamber. Since NC will easily dissolve in acetone, directly applying the liquid onto the fiber mats will make the nanosized fibrils dissolve and the network will collapse. However, we have demonstrated that, by using a mild vapor treatment, partial melting of the fibrils without complete collapse of the network could be achieved. The treatment was performed by placing a sample with NC fibers in a chamber together with an open vial containing acetone. The chamber was subsequently sealed for 30, 60, or 90 min. After 30 min treatment, no visual difference was observed. However, after 60 min, the previously white sample had started to become transparent, and after 90 min the sample became completely transparent. NC is inherently transparent, and the white color comes from the scattering of light in the fiber network. Therefore, the gradual transition from white to transparent indicates that the fibrils are fusing, and the network is collapsing. The treatment greatly improved the structural integrity of the NC mats, as can be seen in Figure SI2e (Supporting Information), where a piece of the mat could be lifted from the aluminum support (which was difficult in pristine samples as shown in Figure SI3 in the Supporting Information). Figure 1a illustrates the mechanism of charge transfer in this particular material combination, where NC becomes negatively charged, lignin becomes positively charged, and the conductive carbon induces counter charges on the different electrodes, resulting in a current running through an external load. This device configuration, where two triboelectric layers are put in contact followed by separation in the direction perpendicular to the plane of the layers, is often referred to as the “contact–separation mode.” The other three basic modes, which use different electrode configurations and motions, are called “sliding mode,” “single‐electrode mode,” and “free‐standing mode.”^[^ ^48^ ^]^

**Figure 2:**
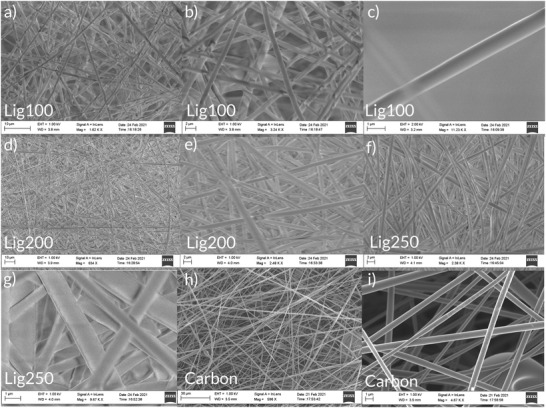
SEM images of lignin films stabilized at 100, 200, or 250 °C (Lig100, Lig200, and Lig250 respectively), as well as carbonized lignin fibers (carbon). a–c) Lig100 sample with scale bar being10, 2, and 1 µm, respectively. d,e) Lig200 sample with scale bars of 10 and 2 µm, respectively. f,g) Lig250 sample with scale bars of 2 and 1 µm, respectively. h,i) Graphitized lignin sample with scale bars of 30 and 1 µm, respectively.

**Figure 3:**
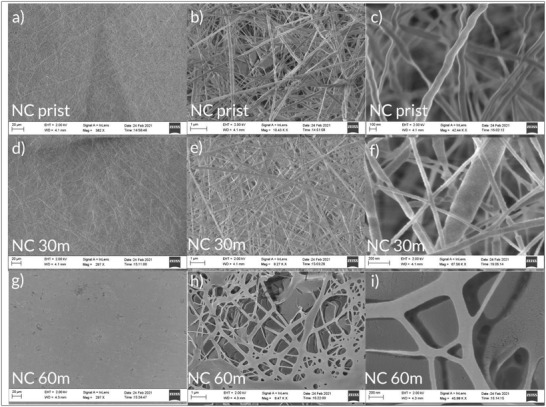
SEM images of pristine NC films (NC prist) and NC films treated with acetone vapor for 30 min (NC 30 m) or 60 min (NC 60 m). a–c) NC prist sample with scale bars of 20, 1, and 100 nm, respectively. d–f) NC 30 m sample with scale bars of 20, 1, and 200 nm, respectively. g–i) NC 60 m sample with scale bars of 20, 1, and 100 nm, respectively.

**Figure 4:**
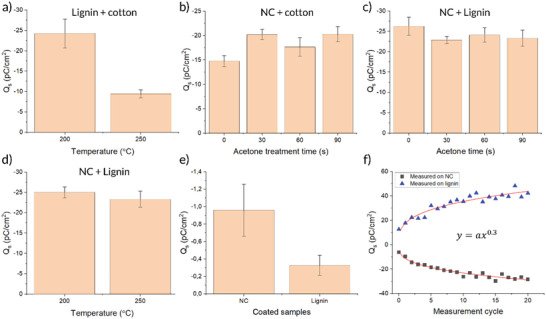
a–e) Surface charge measured on fiber samples with different post treatments after contacting with different reference materials. f) Charge buildup over repeated contact cycles measured on lignin and NC separately. Each mean value and error bars in panels (a)–(e) were calculated from ten individual measurements (*n* = 10), while the data point in panel (f) represents single measured values.

**Figures** **2** and **3** show scanning electron microscopy (SEM) images of the different materials used in the study, with different post treatments (heating and pressing for the lignin and acetone vapor treatment for the NC). **Figure** **4** shows the lignin samples as well as the carbon fiber mats formed by graphitizing the already formed lignin fiber mats. The fibers were shown to have a diameter of ≈1 µm. With increasing stabilization temperature, the fibers were observed to become more densely packed and flatter. The compacting of the lignin fibers explains the improved mechanical strength and the reduction in fiber transfer during contacting. The carbonized fibers retain their circular shape with no sign of damage of degradation. The sheet resistance of the carbon fibers was measured using a four‐probe resistance system and was determined to be 47 Ω sq^−1^. Since TENG devices usually have a high impedance in the MΩ range, the comparatively small resistance of the carbon fiber will not inhibit the device performance.

Figure 3 shows different levels of magnification of pristine NC samples and samples treated with acetone vapor for 30 or 60 min. The NC fibers are small compared to the lignin fibers with a diameter of ≈50–200 nm. When compared to the lignin mats, they have a larger specific surface area. After 30 min of vapor treatment, the sample still show the fiber structure, although they appear to be more densely packed. However, after 60 min of treatment, the top layer of the sample has started to merge. Initially, in Figure 3g at low magnification, it appears that the whole fiber structure has collapsed. However, observing at higher magnification (Figure 3h,i), it is seen that the inner structure is still open and porous, due to some openings which are not fully closed. Here it can be concluded that the fibers have merged into a continuous web with flat filaments.

A surface charge DC voltmeter was used to determine the evolution of the charge density of lignin and NC samples after contact electrification as a result of the different post treatments. Lignin and NC samples were either put in contact with cotton fabric or with each other, after which the surface voltage was measured. The voltage value was further converted to charge density in pC cm^−2^ (calculated using Equation (1) in the “Experimental Section”). The surface charge density depends on several factors such as the specific contact area (in this case, the contact area of lignin and NC fibers), difference in work function of the two contacting materials and the pressure/friction between the surfaces.^[^ ^49^, ^50^ ^]^ The details of the measurements are found in the “Experimental Section.” It should be noted that a relatively small amount of force/friction was applied during these measurements; therefore, the values of charge density reported here are small compared to values in literature.^[^ ^14^ ^]^ However, the purpose of these measurements was to determine the relative trends in charge density when applying different post‐treatment methods to the fiber mats and not to determine the maximum achievable charge density (which can instead be found at a later point in this report).

The surface charge measurements were performed by attaching a reference material (cotton cloth) to a circular weight of 100 g. Cotton is suitable as a reference material as the “zero point” as it sits close to the middle of the triboelectric series.^[^ ^41^, ^51^, ^52^, ^53^ ^]^ The weight was put on top of the sample surface so that the reference material came in contact with the sample. This was done without applying any additional pressure besides that applied by the weight. After removing the weight, the surface charge on the sample was immediately measured using a surface voltmeter. Figure 4a shows the surface charge of lignin samples stabilized at different temperatures. As was shown in Figure 1g, the lignin stabilized at 100 °C (Lig100) was very fragile and would come off in large pieces when contacted to another surface (like cotton or NC). On the other hand, the lignins stabilized at 200 °C (Lig200) and 250 °C (Lig250) were strong enough to survive the measurement without significant transfer of fibers. It is clear that both Lig200 and Lig250 acquire a negative charge with respect to cotton, thereby placing themselves below the zero point in the triboelectric series. Lower charge density was recorded for Lig250 when compared to Lig200. The reason for this can be either because of a chemical modification (e.g., oxidation by the thermal treatment), which shifts the lignin material further up in the triboelectric series, or by a reduced contact surface area. Since the SEM images in Figure 2 showed a compacting and flattening of the lignin fibers at elevated temperatures, the latter is perhaps the more plausible explanation. We can thereby conclude that there is a tradeoff between mechanical and electrical properties when performing the heat‐induced cross linking. Future work should investigate ways of improving the method to achieve good mechanical stability without loss in contact electrification. Figure 4b,c shows the charge on NC samples treated with acetone vapor for different lengths of time (0, 30, 60, or 90 min; where 0 min means untreated samples) using either cotton or lignin fibers (Lig250) as the reference material. In both cases, NC acquires a negative charge, thus putting NC below both lignin and cotton in the triboelectric series. Even though there should be a larger gap in the triboelectric series between NC and cotton compared to NC and lignin, a larger charge was still built up in the NC–lignin combination compared to the NC–cotton combination, suggesting that the surface area plays a predominant role in these material combinations rather than the work function difference (or possibly a combination of the two). An increase in charge density was observed in acetone‐treated samples when using cotton as a reference, while the opposite was observed when using lignin as the reference. This difference in the effect of the post treatment is difficult to explain by the relative positions in the triboelectric series alone. Instead, it points to a more complex relationship between the different fiber structures of lignin, NC, and cotton. It is possible that the enhanced mechanical stability leads to less damage upon contact, therefore, an improved charge transfer, but also a loss in specific contacting surface area which leads to a reduction in charge transfer. These two properties might influence to different degrees depending on the reference material, resulting in the apparent contradictory trends. In both cases, treatment beyond 30 min did not result in significant further change, suggesting that it is primarily the top layer of the sample (which would be affected fastest by the vapor treatment), which is affected by the treatment, while the bulk remains open and porous (as was previously suggested based on the images in Figure 3).

Figure 4d shows the charge on pristine NC samples when using lignin stabilized at 200 and 250 °C as counter material. A small reduction in the mean charge density can be seen when using the lignin sample treated at the higher temperature, although, with the overlapping error bars, this difference is not significant. Therefore, the high‐temperature stabilization can be seen as more favorable when considering the improved mechanical stability.

To compare the effect of the microstructure of the fiber mats, thin and flat samples of lignin and NC were prepared on paper and cotton substrates. The samples were prepared by formulating the materials into liquid solutions/dispersions, which were then coated on the substrates (see the “Experimental Section”). Figure 4e shows the charge density, versus cotton, of the printed samples of lignin and NC. The flat samples showed 1–2 orders of magnitude lower charge densities compared to their fiber mat counterparts. For the NC sample, the charge density was 21 times higher (−20.30 pC cm^−2^) for the electrospun fibers with 90 min acetone treatment compared to the coated NC (−0.96 pC cm^−2^). For the lignin fibers, the charge density was 73 times higher (−24.23 pC cm^−2^) compared to the coated lignin samples (−0.33 pC cm^−2^). The large difference in charge density between the coated samples and the electrospun samples illustrates the importance of the nanostructuring of the triboelectric layers. Figure 4f shows the charge on lignin and NC samples, respectively, after contact with each other over repeated cycles. It is evident that the charge builds up more and more with repeated cycles, following a power law with an exponent of 0.3. The same charge accumulation was not observed in the flat printed samples, suggesting that the fibers are accumulating charge which travels into the bulk of the fiber mat and becomes trapped. This type of 3D charging has been observed in previous publications using porous electrodes.^[^ ^54^ ^]^

**Figure 5:**
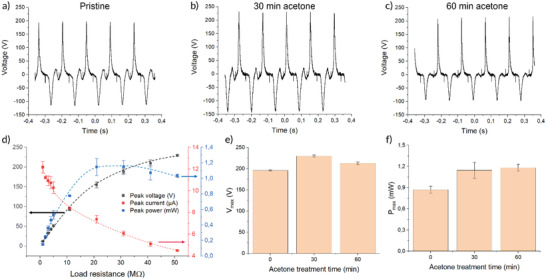
Triboelectric response from F‐TENG in an automated actuator setup. a–c) Voltage response for F‐TENG using Lig250 and NC with different acetone treatments (no treatment, 30 min, or 60 min). d) Voltage, current, and power of F‐TENG from Figure 3b using different load resistor values. e,f) Maximum achieved voltage and power for the F‐TENGs in Figure 3a–c. (*n* = 5 for Mean value and SD calculations in panels (d)–(f)).

**Figure** **5**a–c shows the voltage response of F‐TENG electrodes, assembled using Lig250 and NC (with different degrees of acetone vapor treatment), measured in an automated actuator setup at 7 Hz frequency (see the “Experimental Section” for information on the actuator setup as well as Figure SI4 in the Supporting Information). The material combination using the NC mat treated for 30 min with acetone vapor showed the largest peak‐to‐peak voltage (*V*_p–p_) of ≈375 V. Figure 5d shows the voltage, current, and power of the material combinations from Figure 5b while varying the load resistor value between 1 and 51 MΩ. The highest generated power was observed when applying a load resistance of 20 MΩ, which is consistent with the previously reported TENG having a maximum power in the same range.^[^ ^55^ ^]^ This resistance value also reveals the internal impedance of the F‐TENG, since the highest power output will be reached when the impedance of the device is matched with the external load. The voltage and power values are comparable to other works using biobased materials on both the triboelectric layers. Chen et al. used crepe cellulose paper in combination with nitrocellulose membranes and could achieve 196.8 V and 16.1 W m^−2^.^[^ ^28^ ^]^ Shi et al. likewise used cellulose paper in combination with nitrocellulose and could achieve 60 V and 0.83 W m^−2^.^[^ ^31^ ^]^ Figure 5e,f shows the maximum recorded values for the three devices in Figure 5a–c. While the 30 min treated sample had the highest maximum voltage (as well as the highest *V*_p–p_), the sample treated for 60 min had a slightly larger average power, although the error bars of the two are overlapping. **Table** **1** summarizes the main material components (triboelectric layers and electrodes) as well as the maximum electrical performance (voltage, current, and power) for this work and several references using at least one material component solely composed of biobased materials. As can be seen, the F‐TENG presented in this work performs well with respect to the other references, while being the only device demonstrated using only biobased materials. It is also one of the few examples of using lignin as a triboelectric material, and to the best of our knowledge, it is the only report using pure lignin. The closes example is Bao et al., where a combination of lignin and starch was used as the positive triboelectric layer together with Kapton and copper.^[^ ^37^ ^]^ The lignin F‐TENG in this work showed 66 times higher voltage, 400 times higher current, and 800 times higher power compared to the lignin–starch TENG.

**Table 1:** Comparison of materials and triboelectric performance of earlier works using biobased materials in TENG

Reference	Negative tribo‐layer	Positive tribo‐layer	Charge collector	Max *V* [V]	Max *I* [µA]	Max *P* [W m^−2^]
This work	Nitrocellulose	Kraft lignin	Bio‐carbon	232	12	1.6
[28]	Nitrocellulose	Cellulose	Copper	197	32	16.1
[31]	Nitrocellulose	Cellulose	CP + paper	60	9	0.8
[57]	FEP	Cellulose	Copper	286	4	0.43
[37]	Kapton	Lignin + Starch	Copper	3.5	0.03	0.002
[26]	PVDF	Cellulose	Aluminium	7.9	5.1	0.1
[27]	FEP	Cellulose + silver	Aluminium	650	11	1.75

**Figure 6:**
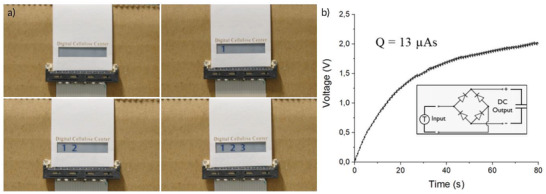
F‐TENG driving electronic devices and charging a supercapacitor. a) Photographs of an electrochromic display on cardboard being powered by an F‐TENG. b) Voltage of a 7.5 µF supercapacitor while being charged by an F‐TENG to a total charge of 13 µA s.

To demonstrate the feasibility of using all bio TENG to power electronic systems, the F‐TENG materials were used to power a printed electronic device, namely an electrochromic display, with pixels in the form of numbers 1, 2, and 3. **Figure** **6**a shows the display pixels turning ON one by one when running the actuator setup at 7 Hz frequency. The printed electrochromic display pixels consist of two electrodes (the conductive polymer poly(3,4‐ethylenedioxythiophene):polystyrene sulfonate (PEDOT:PSS) and carbon) and an electrolyte.^[^ ^56^ ^]^ This is also the structure of a supercapacitor, and indeed, the pixels also function as small supercapacitors, with charges accumulating in the conducting polymer which causes the color to change from transparent to blue. The charging of the display therefore also shows that the power generated by the F‐TENG can be stored in printed and flexible energy storage devices. Figure 6b shows the charging curve when powering one of the display pixels (the number 3). A diode bridge (shown in the inset of Figure 6b) was used to produce a DC voltage from the F‐TENG. The capacitor was charged to 2 V in 80 s with a total charge of 13 µA s, and a capacitance of 6.5 µF. Figure SI5 (Supporting Information) shows the chronoamperometric charging and discharging to 2 V. The discharge curve was integrated to determine the capacitance of the pixel. The reason for the capacitor not reaching higher voltage values than 2 V, even though the F‐TENG has been shown to produce several hundred volts, is because of the electrochemical nature of the device. Any electrochemical system containing water will be subjected to side reactions, like water hydrolysis, at elevated voltages. Therefore, this particular energy storage system was limited to ≈2 V. To store the full voltage generated by the F‐TENG, a dielectric or electrolytic capacitor could be used. However, these capacitors generally have a much lower capacitance per unit area, and these are more difficult to produce and integrated into flexible systems, e.g., by printed electronics.

With the charging of the conductive polymer‐based supercapacitor, it is possible to estimate the amount of transferred charge from each pressure cycle. 13 µC (or µA s) was stored over 80 s at a frequency of 7 Hz, which is equivalent to 560 pulses. This gives us 23.2 nC per pulse, which (with a sample diameter of 3 cm) is equivalent to 33.14 µC m^−2^ per cycle. By further taking half of this value, we can estimate the charge transfer upon contact, similar to the measurements in Figure 4. Here we get the value ≈16 µC m^−2^, a value which is much greater than those recorded using the surface charge voltage meter, and that is similar to values in literature for material such as wood.^[^ ^14^ ^]^ As was pointed out earlier, this difference in magnitude is likely caused by the very small forces applied on the samples in the measurements of Figure 4.

**Figure 7:**
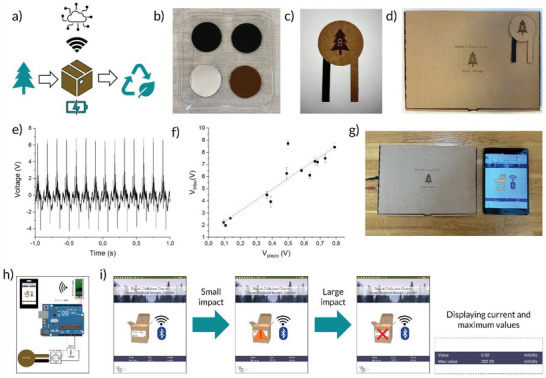
F‐TENG device in a smart package application. a) Schematic of the smart package concept, b) photographs of the F‐TENG triboelectric layers and electrodes, c) photograph of an F‐TENG device, d) photograph of the smart package, e) voltage output of the F‐TENG with a 51 MΩ load resistor, f) F‐TENG voltage versus piezovoltage (*n* = 5 for mean value and SD calculations), g) photograph of the smart package and a tablet displaying the App. h) Schematic of the smart package electronics, i) screenshots from the app window during operation.

Finally, a fully assembled F‐TENG device was constructed and demonstrated in a smart package application (illustrated in **Figure** **7**a). As the use of mail‐order services is rapidly increasing, there is a drive to develop the so‐called smart packages with built‐in electronic functionality, which makes it possible to monitor and track the history of how the package has been handled during transport. Parameters of interest can be temperature, humidity, acceleration, and impact to name a few. This is especially important for packages containing expensive goods, as well as medically critical goods like the transport of donated organs and the distribution of vaccines. However, by adding electronics to the otherwise mainly biobased (cardboard and paper) packages, we are adding to the ever‐increasing problem of e‐waste and plastic waste. Hence, the manufacturing of electronic systems from biobased materials that can be recycled in the same way as the cardboard boxes could mitigate this problem. For this reason, the F‐TENG is ideally suitable for single/short time use products with built‐in electronic intelligence.

Figure 7b shows a photograph of the main device components: the triboelectric layers (nitrocellulose and lignin fiber mats) and the electrodes (conductive graphitized lignin fiber mats). The assembled device, shown in Figure 7c, uses cardboard as the support and encapsulation material, as well as a spacer made from paper, which is used to separate the triboelectric layers. The cardboard contacts were coated with a nanocellulose‐based carbon paste to connect the device with the measurement apparatus. The step‐by‐step assembly of the device is shown in Figure SI2 (Supporting Information), and the experimental details can be found in the “Experimental Section.” A photograph of the smart package can be seen in Figure 7d. The voltage output of the F‐TENG device when placed in the actuator setup is shown in Figure 7e, and the device in operation while tapping on it with a finger can be seen in Video S1 (Supporting Information). The device generates few volts when connected to a 51 MΩ load at 7 Hz. The considerably lower voltage of the device compared to the values measured in Figure 5 can have several explanations. First, the device had an outer diameter of 6 cm while the triboelectric layers had a diameter of 4 cm. The device was too large to be properly centered in the actuator setup, consequently the movable arm would hit the device off center, and therefore the maximum amount of force was likely not transferred (see Video S2, Supporting Information). However, the more likely explanation is that the support material (cardboard) and the spacer (paper) were stiff, limiting the transferred force and the contact–separation movement of the device. More work will be needed to optimize the device structure and the material properties of the support in order to reach the full potential of the triboelectric materials.

A few volts are however more than enough for the device to be used as a self‐powered impact sensor in a smart package application. For this type of application, it will be important that the TENG has a linear voltage response with respect to the applied pressure, so as to determine the magnitude of forces the package has been subjected to. The actuator setup was equipped with a commercial piezoelectric pressure sensor, which was placed behind an adhesive pad onto which the samples were mounted. This enabled us to measure relative pressure by recording the voltage of the piezoelectric sensor and was used to ensure that each measurement was performed under similar conditions. By varying the position of the movable sample holder relative to the actuator arm, the impact pressure could be changed, which was detected as a change in the piezoelectric voltage. Figure 7f shows the voltage response of the F‐TENG and the piezosensor measured simultaneously. Except for one outlier point, the tribovoltage is linear with respect to the piezosensor (see Table SI1 in the Supporting Information for the linear fit data). Since the commercial piezosensor has a linear voltage response with respect to applied pressure, the F‐TENG must also have a linear response. A setup capable of more precisely measuring pressure should be used in the future to determine the exact voltage–pressure relationship of the F‐TENG, but since the device is only a prototype with needed for optimization, this is outside the scope of this work. Figure 7g shows a photograph of the smart package next to a tablet displaying the Smart Package App. A more detailed description of the App operation can be found in Figure SI6 (Supporting Information). Figure 7h shows a schematic of the Smart Package electronics, and Figure 7i shows screenshots of the App during operation. In the App window, a package can be seen, as well as a bar showing current and maximum recorded voltage values from the F‐TENG, which was mounted beneath the box. When the voltage value exceeds a certain level, a warning signal will appear. And when exceeding a certain maximum voltage, a cross will appear to indicate possible damage to the package. The full operation of the Smart Package Application can be seen in Videos S3–S6 (with descriptions in the Supporting Information).

## 3 Conclusions

In this work, we constructed, for the first time, a fully biobased TENG (F‐TENG) using lignin, chemically modified cellulose (nitrocellulose), graphitized lignin, paper, and cardboard. The triboelectric layer as well as the conductive layers was nanostructured using electrospinning to enhance the active surface area, as well as to give elasticity to the system. The nanofiber networks had up to 100 times greater charge accumulation capacity compared to dense electrodes, and they displayed a “bulk charge storage,” where charges would gradually accumulation over each pressure‐release cycle. To improve the mechanical stability of the networks, different post treatments were employed to cross‐link the fiber mats. A novel acetone vapor treatment was used for the nitrocellulose fibers, which would dramatically improve the fiber integrity without any significant reduction in charge accumulation. In fact, for some cases the acetone treatment would result in enhanced voltage output. The triboelectric materials were characterized separately and together, and displayed excellent voltage (232 V), current (17 mA m^−2^), and power (1.6 W m^−2^) characteristics, with values comparable to, or greater than, existing literature using similar material systems. This work is also one of the few examples of the use of lignin in TENG devices, and, to the best of our knowledge, the only example of using pure lignin in one of the electrodes. Since lignin is an abundant, but largely untapped natural resource, the use of this biopolymer is a step toward greener electronics solutions. The power generated from a small disk (3 cm diameter) of the triboelectric materials could turn on a printed electrochromic display and charge a 6.5 µF capacitor in 80 s. Finally, a fully assembled F‐TENG device was used as a self‐powered impact sensor in a smart package application. The sensor showed a linear voltage response with applied pressure and could send the information by wireless communication to an in‐house developed App. Since the impact sensor contains only biobased material, there is no need to separate the device from the cardboard package, and the whole system is recyclable. The work shows the feasibility of constructing fully green and sustainable electronics components using biobased materials like wood.

## 4 Experimental Section

### Materials

Nitrocellulose (Protran) was purchased from Amersham. Softwood kraft lignin from the LignoBoost process was provided by RISE Bioeconomy and Health; dispersion‐based pressure‐sensitive adhesive (KIWOTHERM D123) was purchased from KIWO; the nanocellulose‐based conductive carbon ink was prepared by RISE Research institutes of Sweden, and the method of preparation was previously reported.^[^ ^58^ ^]^

### Fiber Mat Preparation

The methods for electrospinning and conversion to carbon fibers were as previously reported.^[^ ^45^, ^46^, ^47^ ^]^ In brief, high molecular weight fraction of softwood kraft lignin was dissolved in dimethyl formamide at a concentration of 47 wt%. Electrospinning was performed at a voltage of 17 kV (generated by a Glassman high‐voltage power supply), a feeding rate of 0.5 mL h^−1^, and a needle‐to‐collector distance of 17 cm. The lignin fibers were thermostabilized by heating from the room temperature to the selected temperatures (100, 200, and 250 °C) at a rate of 0.5 °C min^−1^ and then holding for 30 min at the final temperatures in a MTI Box Furnace (KSL‐1200X) under an air flow of 10 L min^−1^. Lignin fibers were placed between two carbon felts and compressed by placing a ceramic plate with the weight of 2 kg on them during the stabilization. The stabilized fibers were then carbonized in an MTI tube furnace (OTF‐1200X) under a nitrogen flow of 0.3 L min^−1^, by heating from room temperature to 600 °C at a rate of 3 °C min^−1^, holding for 5 min at 600 °C, heating from 600 to 1000 °C at a rate of 5 °C min^−1^, and holding for 20 min at the final temperature.

Nitrocellulose fiber mats were prepared using a similar procedure. A mixture of THF and DMF was used as solvent. The ratio of tetrahydrofuran (THF) and dimethylformamide (DMF) was 3:2 v/v and concentration of nitrocellulose was 10%.

Reference samples of NC and lignin without nanostructuring were prepared by formulating the materials into liquid solutions/dispersions and coating them onto different substrates. The NC was formulated into a screen‐printable ink using dipropylene glycol as solvent (40 mg mL^−1^). The ink was subsequently printed onto paper substrates. The lignin could not easily be coated in the same way. Attempts of making coatings using dissolved lignin resulted in films that were too brittle and would easily crack. Instead, an aqueous suspension of lignin particles was prepared (4 mg mL^−1^) using ultrasonication for 5 min. The suspension was casted onto cotton fabric (the same fabric as was used as a reference material in the triboelectric characterization) to achieve a lignin grammage (≈0.75 mg cm^−2^) closely matching that of the printed NC samples. The particles would not form a continuous coating as with the printed NC samples. However, by using cotton as a scaffold for attaching the particles, the exposed substrate would not interfere with the measurement results as it was the same as the reference material.

### Fiber Mat Cross‐Linking

The lignin fiber mats were cross‐linked by heating them to different temperatures (100, 200, or 250 °C) for 30 min in an oven. NC fiber mats were cross‐linked by acetone vapor by placing them in an enclosed chamber with an open vial of acetone. The treatment was performed for 30, 60, 90, or 120 min.

### Scanning Electron Microscopy

Morphological and structural investigations were performed by using SEM, Sigma 500 Gemini. The samples were coated with a thin layer (5–8 nm) of gold (using IKE thermal evaporator, Balzers, model BA 510) to avoid surface charging of nonconductive samples. It helps samples surface to conduct evenly, and it offers a uniform conductive surface for high‐resolution imaging and structural analysis.

### Surface Charge Measurements

Surface charge measurements were performed using a Surface DC Voltmeter (Model SVM2, AlphaLab Inc.). Measurements were performed by contacting the sample surface with a reference material while applying a constant pressure (100 g weight). After removing the reference material, the surface voltage (*V*) was measured at a distance (*L*) from the sample with the diameter (*D*). The equation below was used to determine the surface charge (*Q*_s_)

(1) $$\begin{array}{c} \begin{array}{l} Q_{s}=\frac{Q}{A}=V\times 3.6\times 10^{-14}\times \frac{f}{f-1}\left[C\;{\text{cm}}^{-2}\right],\\ \text{where}\;f=\sqrt{1+\frac{D^{2}}{4L^{2}}}=\sqrt{1+\frac{4.5^{2}}{4\times 2.5^{2}}}=1.35 \end{array} \end{array}$$

### Voltage Measurements

Triboelectric voltage and electrical power measurements were carried out using an in‐house developed setup consisting of a stationary plane (a) and an oscillating arm (b) (Figure SI4, Supporting Information). The stationary plate was mounted over base that could be precisely moved using micrometer screw to adjust the lateral separation between two substrates during contact and separation movements. The oscillating arm was attached to a linear electromagnetic actuator, and the oscillation frequency was controlled by an in‐built function generator of a digital storage oscilloscope (Keysight Infiniivision DSOX2004A). The oscillation frequency was always fixed to 7 Hz sinusoidal waveform. As part of the setup evaluation, it was found that 7 Hz generated the maximum impact force onto the sample; this frequency value is also dependent on the length of the actuator arm. At lower frequencies, the acceleration is lower and therefore the force is lower. But at higher frequencies, the arm would not have time to fully extend and retract, which also leads to a loss of force. For this reason, the frequency was kept at 7 Hz where to actuator operated optimally, and instead adjusted the impact force by the movable sample holder stage. The substrates to be characterized were attached to either or both parts. The reusable sticky glue “Blue tac” was used to hold the substrates at its place during characterization. In case of stationary plane (a), the piezoelectric sensor was placed between stationary plane and Blue tac. This sensor served as an impact sensor, allowing reproducible measurement of F‐TENG at a particular force. The generated voltage of F‐TENG and piezo‐sensor both were measured using DSO. To measure the generated power by F‐TENG, it was possible to incorporate load resistances using a resistor decade box capable of varying values of load resistance

### Device Assembly

The F‐TENG device, depicted in Figure 7c, was assembled in the following steps: 1) cardboard was laser cut to act as support structures; 2) a double sided adhesive (3 M) was attached to the cardboard; 3) nanocellulose‐based carbon ink was painted onto the cardboard with a paintbrush to act as the contact between the active layers of the F‐TENG and the measurement equipment; 4) the conductive graphitized fiber mats were attached to the still wet carbon ink; 5) a few drops of additional carbon ink were used to glue the lignin film to one of the carbon fiber mats; 6) a filter paper was coated on both sides using a commercial pressure‐sensitive adhesive which was then placed between the second carbon fiber mat and the nitrocellulose film; 7) a paper spacer was put around the triboelectric layers; 8) the two halves were laminated to form the final device. Photographs of the assembly steps can be seen in Figure SI2 (Supporting Information).

### Smart Package Electronics

An Arduino Uno was used to signal recording and processing. An HC‐06 Bluetooth module was used for wireless communication between the Arduino and the App on the tablet. The F‐TENG was connected to the Arduino analog input and ground with a 10 MΩ resistance and 100 pF capacitor placed in parallel.

### Statistical Analysis

Mean values and standard deviation were calculated using the software OriginPro 2020 from OriginLab. For Figure 4a–e, the sample size was 10. For Figure 5d–f, the sample size was 5. For Figure 7f, the sample size was 5. No other pre‐processing or statistical analysis of data was performed in this work.

## Conflict of Interest

The authors declare no conflict of interest.

## Author Contributions

J.E. conceived the main idea, performed the surface charge measurements and triboelectric measurements, constructed the F‐TENG device, and created the smart package application. M.Y.M. built the triboelectric measurement setup and assisted in measurements. O.H. produced the fiber mats. N.H.A. performed the SEM measurements. V.B. supervised the project. All authors contributed to the writing and reviewing of the manuscript.

## Supporting information

Supporting Information

Supplemental Video 1

Supplemental Video 2

Supplemental Video 3

Supplemental Video 4

Supplemental Video 5

Supplemental Video 6

## Data Availability

The data that support the findings of this study are available from the corresponding author upon reasonable request.
