# Supplementary material for: A Forest‐Based Triboelectric Energy Harvester
Source: Glob Chall. 2022 Aug 7;6(10):2200058. doi: 10.1002/gch2.202200058 (PMC9581787; doi:10.1002/gch2.202200058)
Supplement: Supplementary file 1 — Supporting Information [file GCH2-6-2200058-s007.pdf]

Global Challenges
Open Access

Supporting Information

for *Global Challenges,* DOI: 10.1002/gch2.202200058

A Forest-Based Triboelectric Energy Harvester

Jesper Edberg, Mohammad Yusuf Mulla, Omid Hosseinaei, Naveed ul Hassan Alvi, and Valerio Beni*

Supplementary Information:

A forest-based triboelectric energy harvester

Jesper Edberg[1,3]*, Mohammad Yusuf Mulla[1,3], Omid Hosseinaei[2,3], Naveed ul Hassan Alvi[1,3], Valerio Beni[1,3]

[1]*RISE Research Institutes of Sweden, Digital Systems, Bio- and Organic Electronics, Bredgatan 35, Norrköping SE-602 21, Sweden.*

[2]*RISE Research Institutes of Sweden, Bioeconomy and Health, SE-114 86 Stockholm, Sweden.*

[3]*Digital Cellulose Center, Bredgatan 35, Norrköping SE-602 21, Sweden.*

**Corresponding author: jesper.edberg@ri.se*

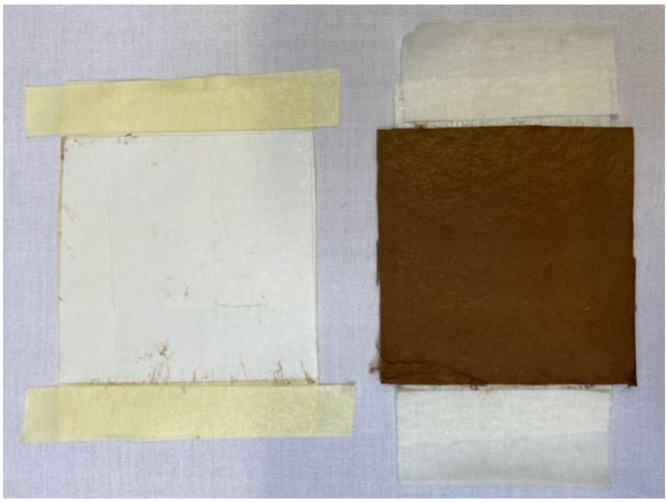

Figure SI1. Photograph of acetone treated nitrocellulose mats (30 minutes treatment) and thermostabilized lignin fiber mats (250 ˚C stabilization temperature) after being pressed together followed by separation. Very little fiber transfer can be observed compared to Figure 1g in the main manuscript.

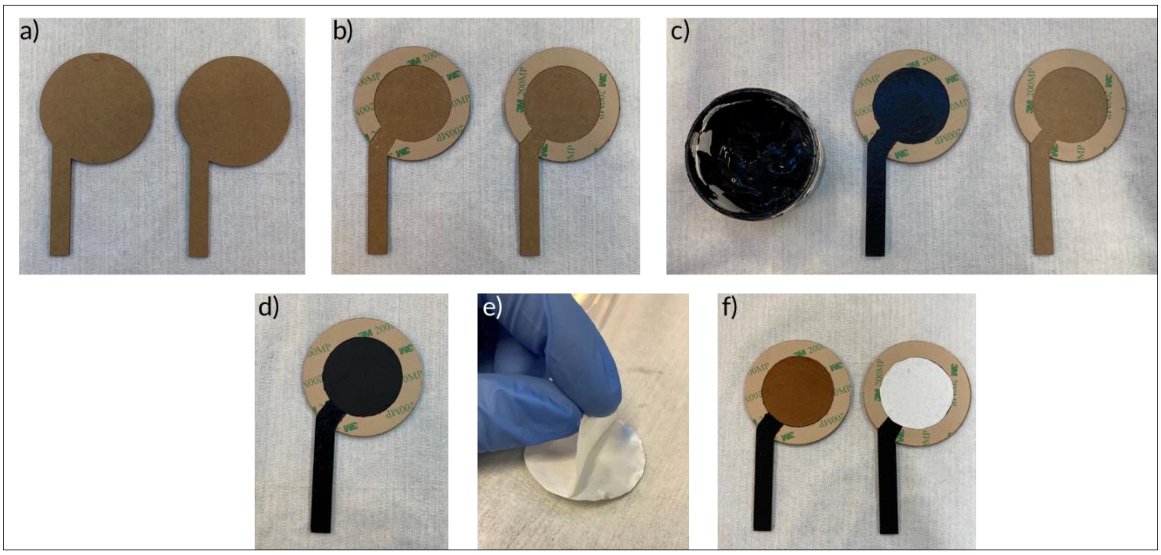

Figure SI2. Photographs of the assembly of an F-TENG device. (a) Laser cut cardboard support structures, (b) double sided adhesive tape, (c) Nanocellulose based carbon ink as electronic contact to the F-TENG, (d) lignin carbon fiber mats attached to carbon ink, (e) acetone vapor treated NC-film peeled from the aluminum support, (f) lignin and NC fiber mats attached to the charge collector.

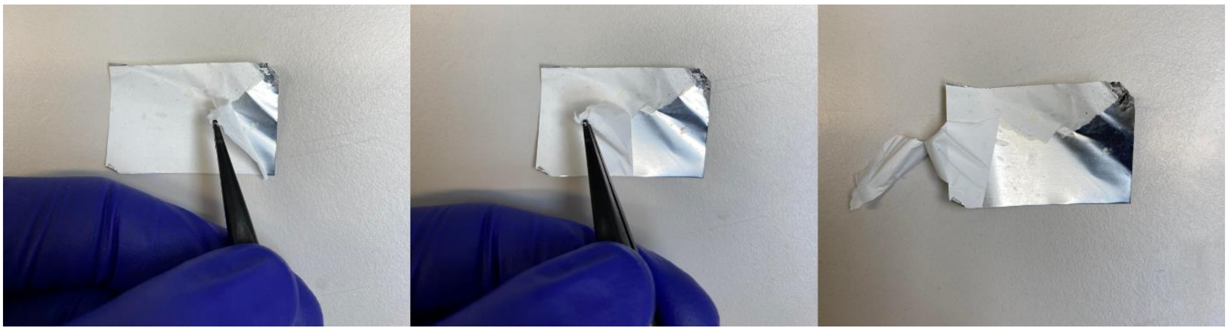

Figure SI3. Photographs of NC fiber mat without acetone treatment being peeled off from the aluminum substrate. The film easily breaks during the peeling process.

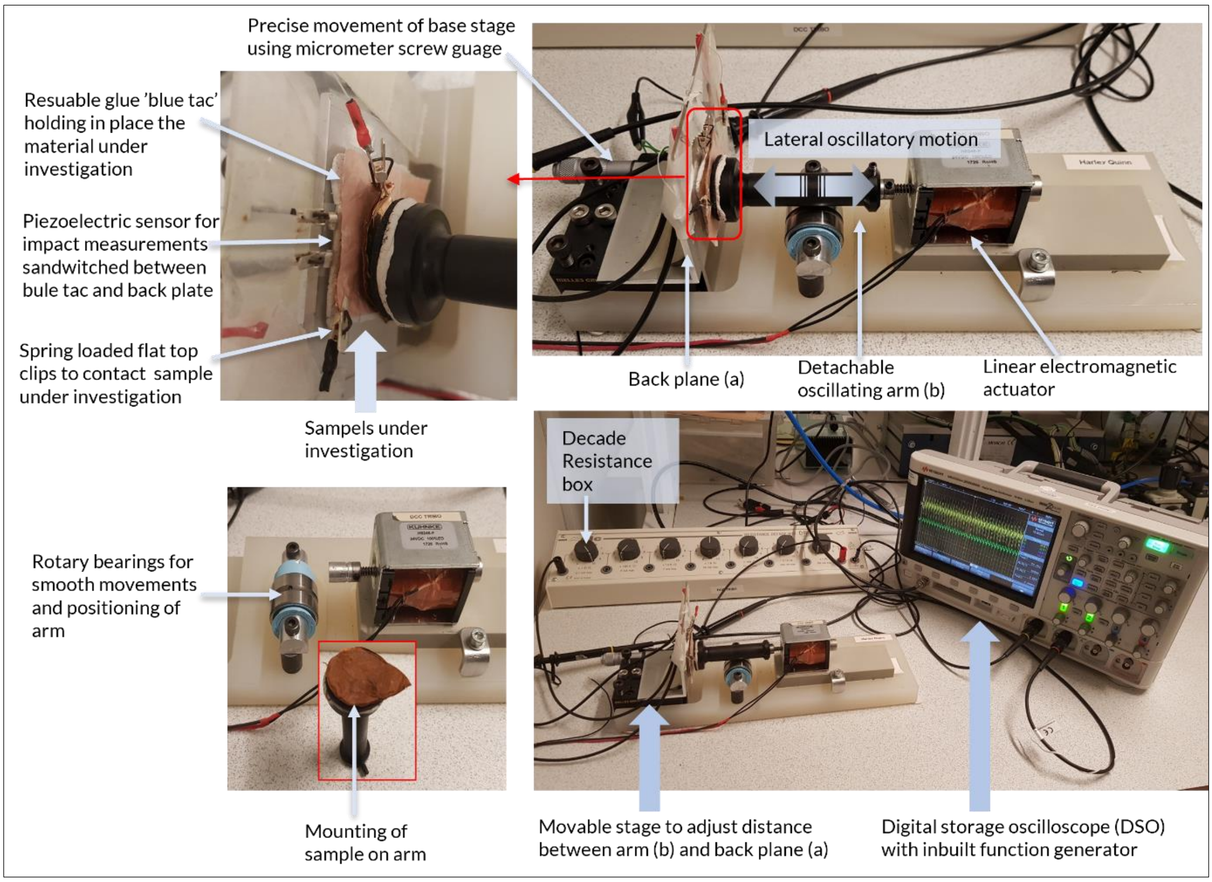

Figure SI4. F-TENG characterization setup. The setup consists of electromagnetic linear actuator controlled by function generator inbuilt into DSO. The resistance decade box allows incorporating load resistance in parallel to the F-TENG for power measurement. The Movable stage with micrometer screw gauge allows precise lateral movement to fix distance between two materials under investigation. DSO allows time-based recording of generated voltage by F-TENG during contact-separation during oscillatory movement of arm.

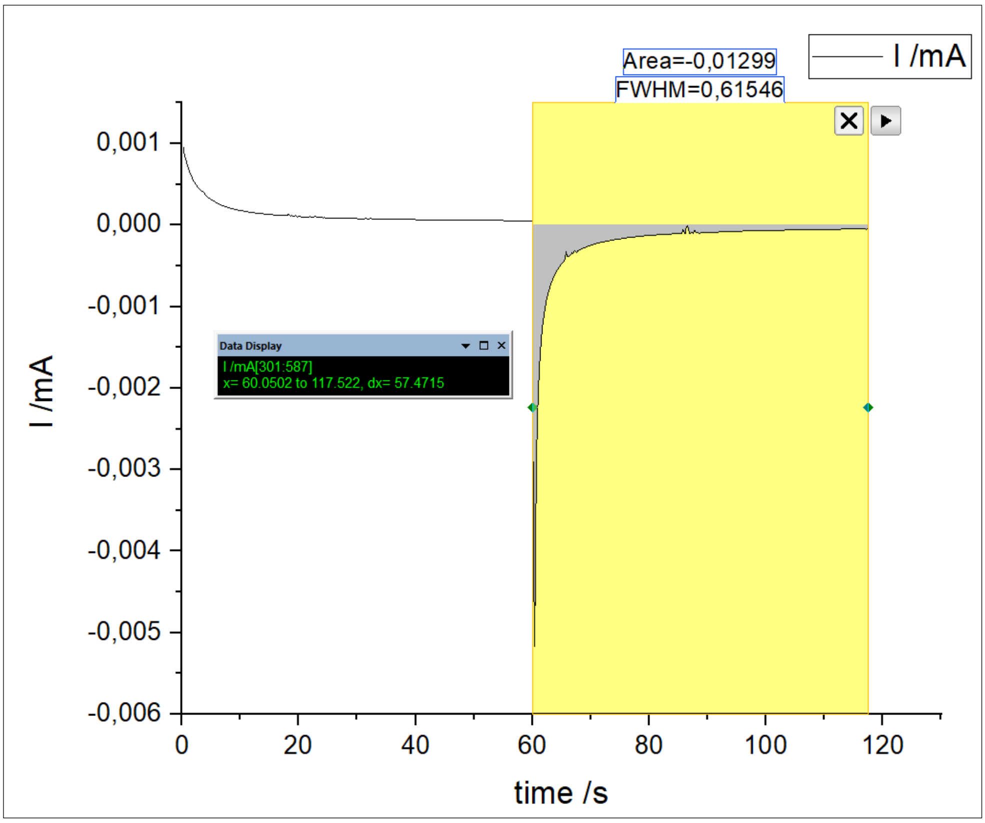

Figure SI5. Charge-discharge curves of electrochromic display pixel measured using chronoamperometry at 2V and 0V respectively. Integrated charge of discharge curve is shown.

Equation	y = a + b*x
Plot	B
Weight	No Weighting
Intercept	1,39966 ± 0,62614
Slope	8,89951 ± 1,18532
Residual Sum of Squares	10,78239
Pearson's r	0,91473
R-Square (COD)	0,83673
Adj. R-Square	0,82188

Table SI1. Linear fitting of the data in Figure 7e.

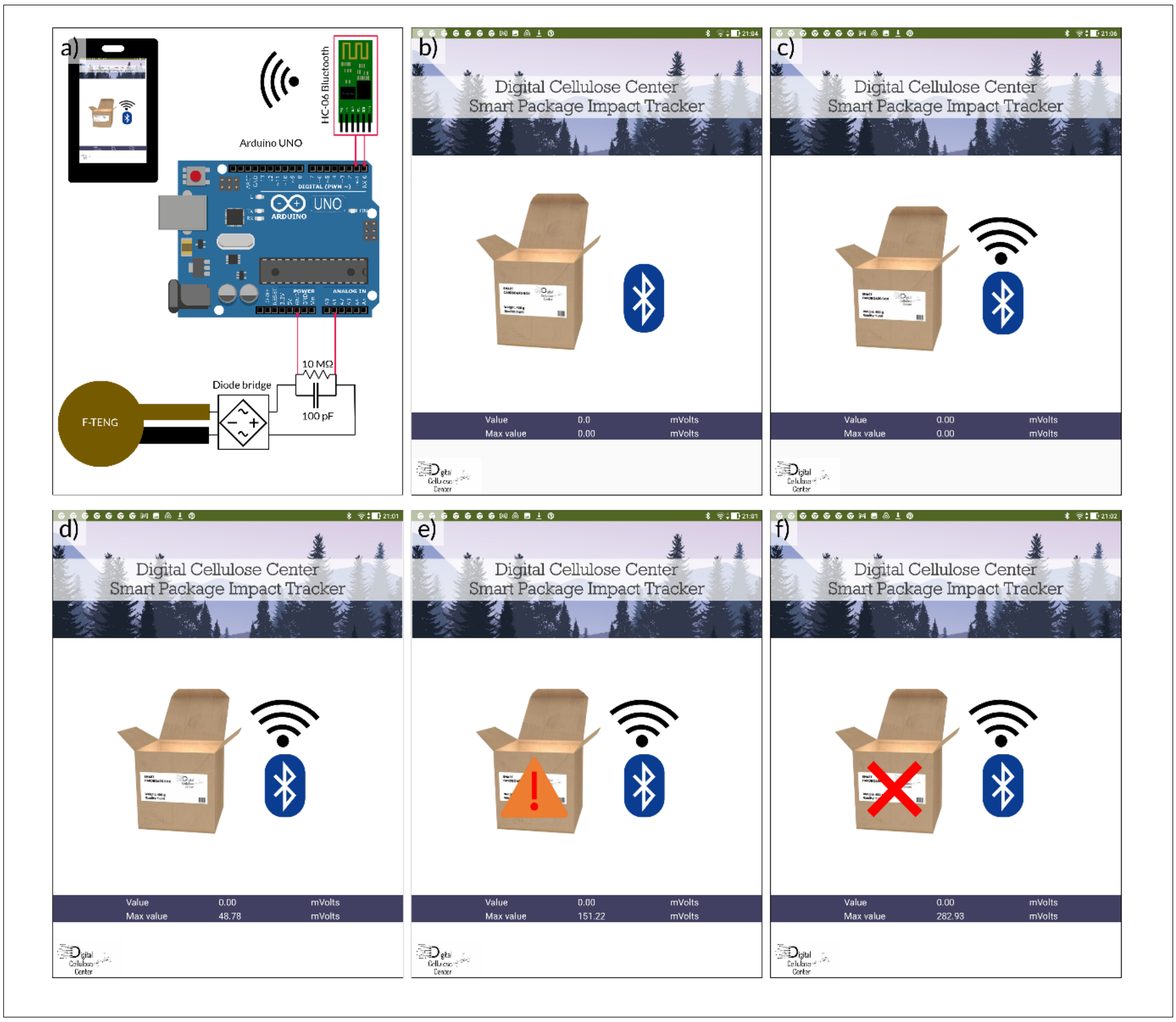

Figure SI6. The Smart Package App. (a) Schematic of the smart package electronics. (b-f) App window during operation.

Operation:

b) Starting window when tuning on the App.

c) Connecting to Bluetooth by pushing the package. A new symbol appears to show that connection has been established.

d) The current and maximum recorded values can be seen in the blue bar.

e) If the voltage signal exceeds a certain value, a warning symbol appears.

f) If the voltage signal exceeds a certain maximum value, a cross symbol appears to indicate possible damage to the package.

By pushing the Digital Cellulose Center logotype in the bottom left corner, the App is reset.

<u>Supplementary Videos 3-6:</u>

SV3: Shows the full operation of the App: Starting the app and connecting to Bluetooth. After dropping the package, the damage-symbol appears.

SV4: The package is dropped, and the warning symbol appears.

SV5: The package is dropped multiple times, and the current Value is updated.

SV6: The App is reset by pushing the Digital Cellulose Center logotype.
